# Topological Relationships Cytoskeleton-Membrane Nanosurface-Morphology as a Basic Mechanism of Total Disorders of RBC Structures

**DOI:** 10.3390/ijms23042045

**Published:** 2022-02-12

**Authors:** Elena Kozlova, Viktoria Sergunova, Ekaterina Sherstyukova, Olga Gudkova, Aleksandr Kozlov, Vladimir Inozemtsev, Snezhanna Lyapunova, Aleksandr Chernysh

**Affiliations:** 1 Laboratory of Biophysics of Cell Membranes under Critical State, Federal Research and Clinical Center of Intensive Care Medicine and Rehabilitology, V.A. Negovsky Research Institute of General Reanimatology, 107031 Moscow, Russia; waterlake@mail.ru (E.K.); vika_23s82@mail.ru (V.S.); olkagood@yandex.ru (O.G.); va.inozemcev@physics.msu.ru (V.I.); snezhanna.lyapunova@yandex.ru (S.L.); amchernysh@mail.ru (A.C.); 2 Department of Medical and Biological Physics, Sechenov First Moscow State Medical University (Sechenov University), 119991 Moscow, Russia; fillnoise@mail.ru; 3 Faculty of Physics, Federal State Budget Educational Institution of Higher Education M.V. Lomonosov Moscow State University (Lomonosov MSU), 119234 Moscow, Russia

**Keywords:** red blood cells, cytoskeleton, membrane nanosurface, structure disorder, atomic force microscopy, hemin, ultraviolet radiation, temperature changes, pH, oxidative processes

## Abstract

The state of red blood cells (RBCs) and their functional possibilities depend on the structural organization of the membranes. Cell morphology and membrane nanostructure are compositionally and functionally related to the cytoskeleton network. In this work, the influence of agents (hemin, endogenous oxidation during storage of packed RBCs, ultraviolet (UV) radiation, temperature, and potential of hydrogen (pH) changes) on the relationships between cytoskeleton destruction, membrane nanostructure, and RBC morphology was observed by atomic force microscope. It was shown that the influence of factors of a physical and biochemical nature causes structural rearrangements in RBCs at all levels of organization, forming a unified mechanism of disturbances in relationships “cytoskeleton-membrane nanosurface-cell morphology”. Filament ruptures and, consequently, large cytoskeleton pores appeared. The pores caused membrane topological defects in the form of separate grain domains. Increasing loading doses led to an increase in the number of large cytoskeleton pores and defects and their fusion at the membrane nanosurfaces. This caused the changes in RBC morphology. Our results can be used in molecular cell biology, membrane biophysics, and in fundamental and practical medicine.

## 1. Introduction

The state of red blood cells (RBCs) and the capabilities of their gas-transport function depend on the morphology and membrane nanostructure [1]. On the other hand, the cell morphology and membrane nanostructure are structurally and functionally related to the cytoskeleton network, the most important component of RBCs [2]. External influences on RBCs, in particular, pharm-chemicals, bacteria, viruses, ionizing radiation, etc., can affect the rheological properties of blood and disrupt the gas-transport function of RBCs [3,4,5,6]. Several studies have shown that changes in RBCs can cause serious complications in the organism [7,8,9]. Today, there is a special emphasis on investigations of RBC structural organization due to the spread of the SARS-CoV-2 virus [6,10]. At the same time, the characteristic changes in RBC morphology are considered as a key link in the diagnosis of diseases [11,12].

Our study considers the influence of a number of biochemical and physical factors on the structural features of the cytoskeleton structure and how changes in the cytoskeleton can manifest themselves at the level of the membrane surface. Agents of different nature were chosen as factors of influence on RBCs. These were biochemical agents: hemin, endogenous oxidation in the anticoagulant-preservative solution during storage of packed red blood cells (pRBCs), changes in the potential of hydrogen (pH), and physical factors: the influence of ultraviolet (UV) radiation and temperature changes of the RBC suspension in the buffer solution. Hemin (hydrochloric acid hematin) is a product of endogenous hemoglobin oxidation in the body; it can disrupt the connection between proteins in the cytoskeleton network [13,14]. Prolonged storage of pRBCs in hermetic bags with the anticoagulant-preservative solution causes defects in RBC membranes, which increase during storage [15,16,17,18]. Changes in the pH value indicate a degree of medium acidification and, therefore, determine the state of RBC membranes. Increasing temperature causes conformational distortions of the RBC cytoskeleton [19]. UV radiation is a physical factor disrupting membrane structure [20,21]. Ultimately, all these factors lead to changes in RBC morphology.

RBCs morphology is highly variable, ranging from normal poikilocytosis to sphero-echinocytes and destruction under anemia and other diseases. The polymorphism occurs at the level of the cytoskeleton, at the level of the membrane nanostructure, and at the level of the cell as a whole. This study is devoted to the investigation of relationships between these structural levels and the mechanisms generating these relationships.

The relationships between cytoskeleton destruction, membrane nanostructure, and morphology were studied using atomic force microscopy (AFM) as the most effective method of visualization and investigation of molecular structures of biological objects [12,22,23].

Our results provide examples of the appearance of large pores in the cytoskeleton and membrane nanodefects under the action of various physical, biological, and chemical nature agents. We have shown how cytoskeleton filament ruptures lead to large pores formation and how this process appears on the membrane nanosurface in the form of grain domains and topological defects.

## 2. Results

In this work, five factors of different nature and different ways of action were used as agents of influence on the morphology and nanostructure of RBC membranes. These were biochemical agents: hemin, endogenous oxidation of the anticoagulant-preservative solution during storage of pRBCs, changes in the pH value, and physical factors: the influence of UV radiation and temperature changes of RBC suspension in the buffer solution.

### 2.1. Hemin

Hemin is protoporphyrin IX containing a ferric iron (Fe^3+^) ion with a coordinating chloride ligand formed by the influence of hydrochloric acid on hemoglobin [24]. Hemin, hydrochloric acid hematin, is endogenously produced in the human organism during the renewal of RBCs. It can be formed as a result of hemolysis, vascular damage, blood loss, and release of hemoglobin into the bloodstream. In these cases, oxidation of heme and production of hemin may occur [25]. In addition, hemin occurs in the stomach under the influence of enzymes and hydrochloric acid of gastric juice. It stains black color the erosion bottom and stomach ulcers and duodenum [26]. The methemoglobin formation increases the probability of hemin appearance in the organism. Proteins in human blood, such as hemopexin and serum albumin, bind to hemin [27].

**Figure 1:**
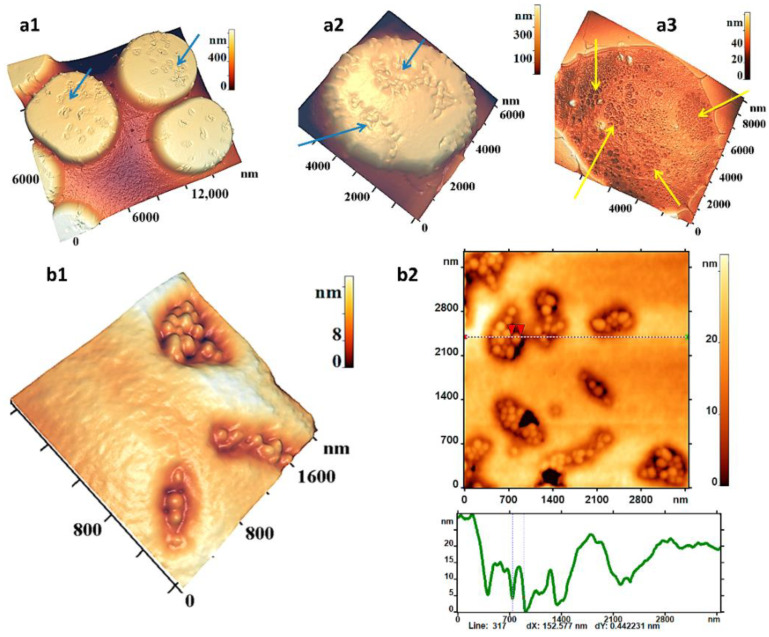
Defects of RBC membranes under the influence of hemin (1.2–1.5) mM, AFM images. (**a1**–**a3**) Defects on cell membrane: (**a1**) on the ensemble of cells, (**a2**) on the single cell, (**a3**) image of ghost (cytoskeleton) of single cell. Blue arrows show domains on the cellmembrane, yellow arrows show domains on the cytosckeleton (**b1**,**b2**) Nanostructure of defects: (**b1**) 3D AFM image, (**b2**) 2D AFM image and its profile. Incubation time is 120–200 min. Size scales and color scales of heights are indicated for AFM images.

Hemin at concentrations of 1.2–1.5 mM formed characteristic defects on the surfaces of RBC membranes in the form of domains containing a number of elementary units-“grains” (Figure 1(a1,a2), blue arrows). The occurrence of such membrane nanostructure defects was shown previously [13]. The sizes of the grains were close and varied between 120 ± 40 nm, and their heights varied between 12 ± 5 nm. The number of grains in the domain could range from 1–3 to 20 or more. We continued our research and showed that domains have different modifications of shapes: from crowded, horseshoe-shaped to almost linearly elongated (Figure 1(b1,b2)). The number of RBCs containing such domains at the indicated concentrations was over 80% in an ensemble of 1500 cells (Figure 1(a1,a2)). Increasing hemin concentration to 2.5–3.0 mM led to an increase in the number of domains (and, accordingly, grains) on the RBC surface and their merging. Moreover, the sizes of individual grains remained practically unchanged. The domains of defects on the membrane surface in the enlarged scale 2000 × 2000 nm^2^ as 2D and 3D AFM image and their profile are shown separately (Figure 1(b2,b1)). Figure 1(a3) shows the AFM image of the ghost of a single RBC after the influence of 1.5 mM hemin. In this image, yellow arrows show fragments that correspond to domains on the membrane surface (Figure 1(a1,a2)). The sizes and shapes of cytoskeleton defects in the ghosts correspond to those on RBC membranes.

**Figure 2:**
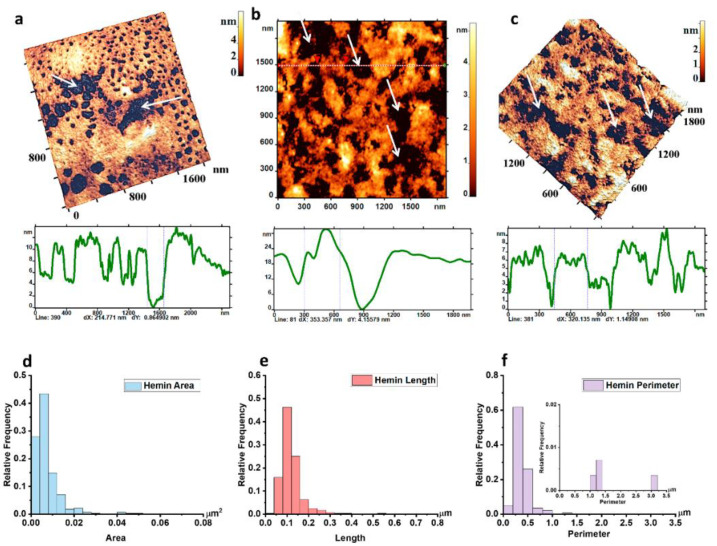
Defects of RBC cytoskeleton under the influence of hemin (1.7 mM, incubation time 120 min). (**a**–**c**) 3D AFM images fragments (1800 × 1800 nm^2^) of cytoskeleton and their profiles, white arrows indicate the appearance of large pores. Size scales and color scales of heights are indicated for AFM images. The cytoskeleton heights did not exceed 5 nm (colored height scales). Relative frequency histograms: (**d**) areas of single pores, (**e**) lengths of single pores, (**f**) pore perimeters; embedded histogram for large pores at the appropriate scale. There are ensemble data of 283 measurements.

The question arises: what occurred in the cytoskeleton structure when the defects appeared on the membrane surface? What is the cause of the appearance of these domains on the membrane? To answer these questions, the cytoskeleton structure of individual RBCs under the influence of hemin at the indicated concentrations and incubation times (Figure 2) was studied in this work. In all presented images, the cytoskeleton heights did not exceed 5 nm (color height scales, Figure 2a–c). This meant that in these images and in the images shown in the figures below, only the cytoskeleton network without side structures was recorded.

Under the influence of hemin, large pores appeared in the cytoskeleton network structure. The length of such pores ranged from 0.20 to 0.54 μm, and the perimeter from 1.5 to 3.02 μm (Figure 2). The diameters of such pores were measured in the range of 350–1300 nm (control pore diameters were 50–150 nm). Obviously, the number of large pores was significantly lower compared to the small (control): the relative frequencies of small (control) pores were 0.6, and large pores were 0.01 (Figure 2f, embedded histogram). The width of the control cytoskeleton filaments was from 30 to 50 nm, and the maximum could reach 353 nm (Figure 2b, profile) or more, depending on the fragment structure.

Increasing the hemin concentration to 3 mM and the incubation time to 180 min led to the appearance of large, up to 1500 nm, ruptured pores on the cytoskeleton, and on the membrane surface, the domains merged into large conglomerates (Figure 1(a2)). The molecular processes of these changes in the cytoskeleton are discussed below.

### 2.2. Storage of Packed Red Blood Cells

**Figure 3:**
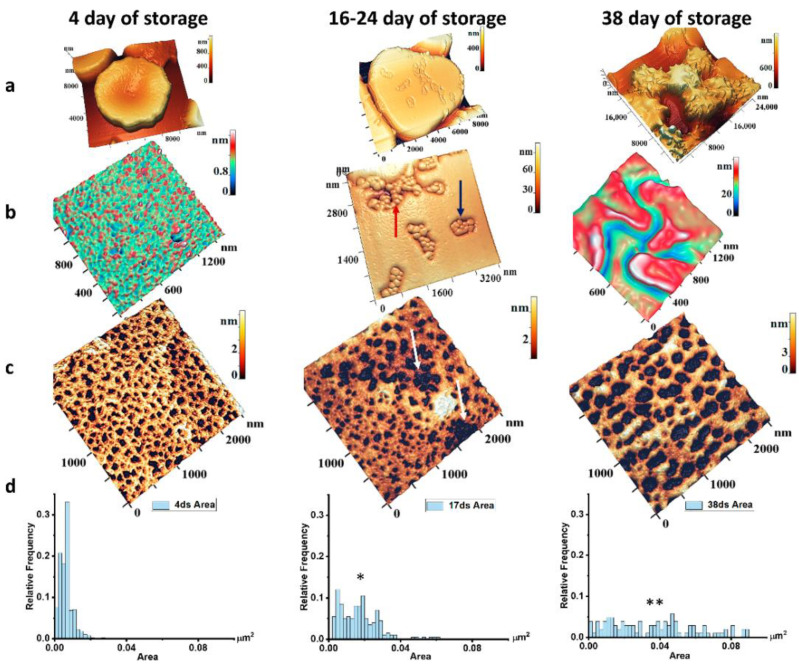
The change of morphology, appearance of RBC membrane defects, and cytoskeleton nanostructure during long-term storage of pRBCs in the anticoagulant-preservative solution. Horizontally: (**a**) RBC morphology; (**b**)membrane nanostructure; (**c**) cytoskeleton nanostructure; (**d**) histograms of relative frequencies densities of individual pores areas. One-way ANOVA test followed by post hoc. Tukey was used: * *p* < 0.05, ** *p* < 0.01 compared to 4 day of storage. Size scales and color scales of heights are indicated for AFM images.

pRBCs were stored for 40 days in hermetic bags with CPD/SAGM in an oxygen-free environment at 4 °C. During storage, the parameters of pRBCs changed as a result of oxidative processes. In particular, the RBC morphology changed, the membrane nanostructure transformed, their stiffness (Young’s modulus) increased. We described some phenomena earlier [15,28]. Now we show that discocytes dominating in the initial period (4 days, 78%) transformed into stomatocytes and planocytes (16–24 days of storage, 64%), and then into echinocytes and spheroechinocytes (38 days of storage, 73%). During the transition period (16–24 days of storage), membrane defects appeared on the membranes of planocytes, similar to those recorded under the influence of hemin (Figure 1). These were domains consisting of a number of grain structures (Figure 3b). First, the domains were simple with a small number of grains, up to 10 (Figure 3b, blue arrow). Then they increased and included more grains (Figure 3b, red arrow). In this period, large pores appeared in the nanostructure of the cytoskeleton network, whose configuration was similar to the membrane domains (Figure 3c, white arrows). This tendency was also observed in the changes of cytoskeleton pore area histograms.

If in the initial period the cytoskeleton pores had areas of 0.01 μm^2^, then on 16–24 days of storage, this value increased by more than 2.5 times, the variance increased, and the maximum values reached 0.06 μm^2^. Cytoskeleton network structure became heterogeneous, conglomerates of large pores appeared. Subsequently, the pores merged, and the cytoskeleton network became as shown in Figure 3c (38 days of storage). Thus, the area variance increased even more, and the maximum pore areas reached values of 0.09 μm^2^ (Figure 3c,d, 38 days of storage).

### 2.3. The pH Value of RBC Suspension in Buffer Solution

One of the main causes of RBC membrane nanostructure disruption is oxidative stress. To modulate changes in morphology and cytoskeleton under different levels of oxidative stress, RBC suspension was placed in the buffer solution with pH of 7.4, 6.8 and 6.4, and each suspension was incubated at 20 °C for 24 h.

**Figure 4:**
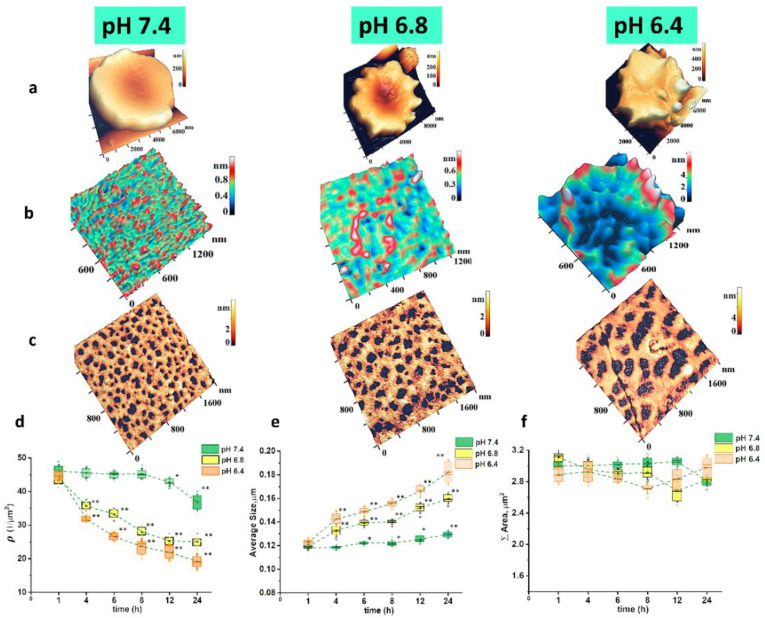
Morphology, membrane nanostructure, and cytoskeleton of RBCs at different pH values of RBC suspension in buffer solution. Horizontally: (**a**) morphology of a single RBC, 3D AFM image; (**b**) membrane nanostructure, 3D AFM image, II order of the spatial Fourier transformation; (**c**) RBC cytoskeleton, 3D AFM image. Incubation time was 24 h. (**d**) Change in the pore density ρ on cytoskeleton for the specified pH values. (**e**) Changes in average pore sizes on a fragment 2 × 2 μm^2^ of the cytoskeleton for the specified pH values. (**f**) Sum of pore areas on cytoskeleton fragment 2 × 2 μm^2^ for the specified pH values. One-way ANOVA test followed by post hoc. Tukey was used: * *p* < 0.05, ** *p* < 0.01 compared to 1 h. Size scales and color scales of heights are indicated for AFM images.

The control values were taken as pH = 7.4, normal physiological conditions. Decreased pH values indicated medium acidification and, therefore, the presence of RBCs in the oxidative stress during the incubation time of 24 h. In the control, there were 78% of discocytes. Along with the decrease in pH, it transformed into pathological forms, echinocytes at pH = 6.8 (65%) and spheroechinocytes at pH = 6.4, (56%) (Figure 4a). The presence of RBCs in solution with pH = 6.8 during 24 h was characterized by the appearance of topological defects in the membrane nanostructure and individual large cytoskeleton pores, which grew and merged (Figure 4b,c). Their average sizes reached 160 nm or more. At pH = 6.4, the topological defects of membrane nanostructure became total, and all cytoskeleton network structures consisted of large pores with sizes up to 200–300 nm (Figure 4b,c). Transformation of the cytoskeleton network developed as the incubation time increased. At pH = 7.4, the pore number (≈45 per μm^2^) and their average size (0.12 μm) practically did not change during 24 h. pH = 6.8, and moreover 6.4 caused a sharp change in parameters: the pore number decreased to 25 and 20, and their size increased to 0.18 and 0.20 μm, respectively, during 24 h (Figure 4d,e). It is important to note that the sum of pore areas on the same fragments (3 μm^2^) during cytoskeleton network transformation remained the same during the entire incubation time (Figure 4f). This meant that the mass of protein filaments stayed the same and only redistributed between cytoskeleton structures.

### 2.4. Temperature

Solution temperature is the most important parameter that greatly influences all structural and functional parameters of RBCs. In this work, we presented the results of changes in the membrane nanostructure and cytoskeleton at temperatures in three ranges. The phase transition temperatures of RBC membranes can vary over a wide range depending on the lipid composition and the conditions in which the membrane is located. We chose those temperature ranges where phase transitions and protein denaturation processes are known to be determined [29]. The first range was 30–37 °C. This range lies above the phase transition temperatures of the broad lipid and cholesterol fraction of RBC membranes and is within physiological limits. The second range was 40–43 °C. This is the range below the phase transitions of cytoskeleton network proteins but lying above physiological limits. The third range was 49–50 °C. This is the range of temperatures when denaturation of the polypeptide chains (αI and βI monomers) of spectrin occurs [30,31].

**Figure 5:**
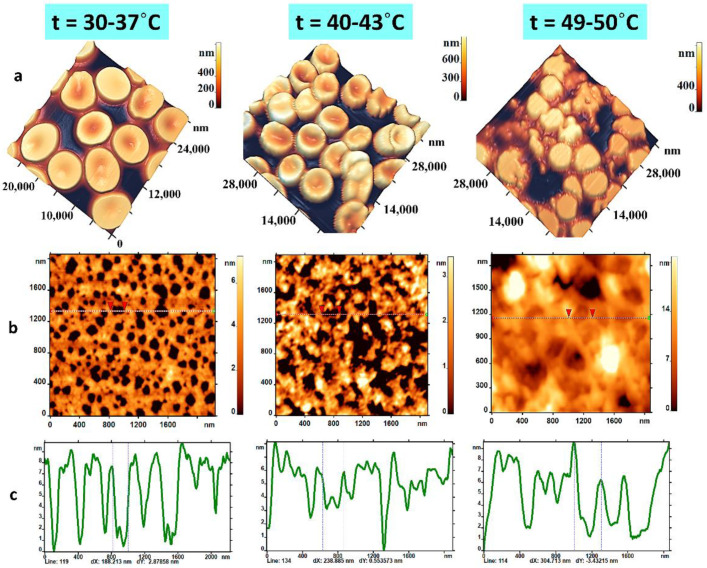
Changes in morphology and cytoskeleton structure at different temperatures of RBC suspension in buffer solution. Horizontally: (**a**) AFM images of RBC morphology; (**b**) transformations of cytoskeleton network in 2D image; (**c**) their profiles at buffer solution temperatures 30–37, 40–43, and 49–50 °C. Incubation time was 60 min. Size scales and color scales of heights are indicated for AFM images. The red triangles show size of a single pore.

At temperatures of 30–37 °C, RBCs were in the form of discocytes (77%), and their cytoskeleton pores were normal size 120–180 nm as shown in Figure 5a–c. Moreover, from 30 to 37 °C, the cytoskeleton network structure practically did not change and was close to one shown in Figure 5b. A temperature increase to 43 °C resulted in the formation of large pores of 300–400 nm on the RBC cytoskeleton. Initially (up to 15 min), these pores were single, but then, by the end of 60 min, more than 70% of the area was covered by the pores. Further increase in temperature up to 50 °C caused melting of RBCs (Figure 5a), and its individual fragments were no longer distinguishable on the cytoskeleton structure (Figure 5b).

### 2.5. Ultraviolet Radiation

In this work, the UV emitter with a wavelength of λ = 254 nm, photon energy E = 4.8 eV, and energy flux Φ = 5.2 W were used. The irradiation area of RBC suspension in the cuvette was S = 12 cm^2^. The suspension was mixed by a magnetic stirrer. The irradiation time was 30 and 60 min.

**Figure 6:**
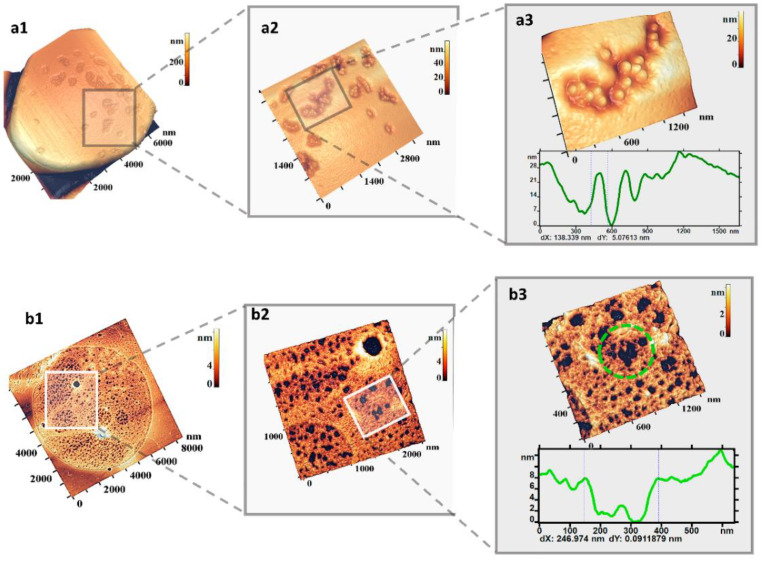
Defects of RBC membranes under the influence of UV radiation. (**a1**–**a3**) Defects on RBC surface and their fragments. (**b1**–**b3**) Cytoskeleton network and its individual fragments. Irradiation time was 60 min. Size scales and color scales of heights are indicated for AFM images.

The surface of RBC membranes under UV irradiation was covered by granular domains (Figure 6(a1–a3)), which were similar in configuration to the defects under the influence of hemin (Figure 1) and during pRBCs storage (Figure 3). Such single domains appeared when the RBC suspension was irradiated for 30 min. Increasing the irradiation time up to 60 min led to the growth of single domains and their fusion on the membrane surface (Figure 6(a2)). Simultaneously, the cytoskeleton network was transformed (Figure 6(b1–b3)). Individual large pores of about 300 nm and more appeared on the network and merged into larger cells up to 600–800 nm in size. The sizes and shapes of large pores of the cytoskeleton network were similar to membrane nanodefects, i.e., the shape and size of the domains.

### 2.6. Topological Relationships “Cytoskeleton Structure–Membrane Nanosurface Structure-RBC Morphology”

**Figure 7:**
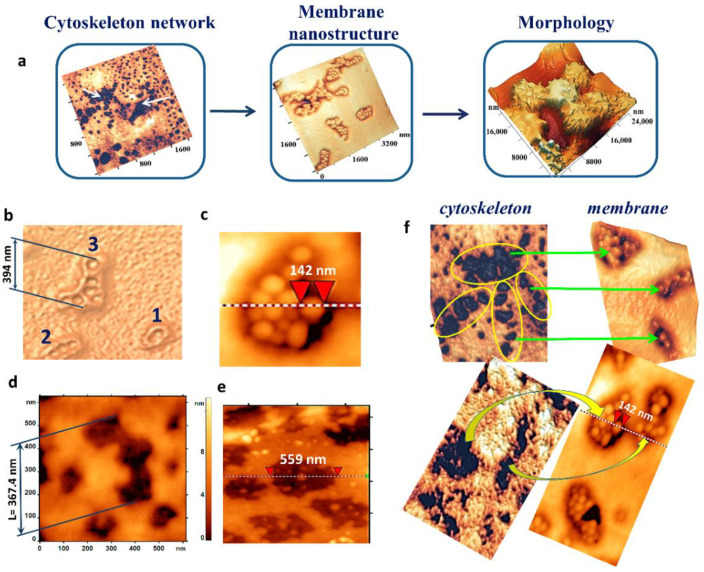
Cytoskeleton pores, membrane nanodefects, and RBC morphology under the action of physical and chemical factors. (**a**) Cytoskeleton pores cause membrane nanodefects on the nanosurface and alterations in RBC morphology. (**b**) Nanodefect fragments on membrane surface; 1—domain consisting of 2 grains; 2—domain consisting of 4 grains; 3—domain consisting of 6 grains. (**c**) Large domain, size of one grain is shown. (**d**) Large pore on cytoskeleton fragment surrounded by small pores. (**e**) Large pores on cytoskeleton fragment. (**f**) Topological relationship of cytoskeleton pores (left) and membrane nanosurface (right). The red triangles show size of one grain.

As a result of oxidative processes caused by the influence of hemin, storage of pRBCs, decrease in pH value, increase in temperature, UV irradiation, interrelated phenomena characteristic of all types of action occurred in the cytoskeleton structure and on the membrane nanosurfaces (Figure 7a). Individual large pores appeared in the cytoskeleton network under loading doses at threshold values (Figure 7d,e). Under these conditions, single defects in the form of grain domains (Figure 7b,c) or topology defects appeared on the membrane surface (Figure 4b, pH 6.8). Increasing of loading dose led to an increase in the number of large cytoskeleton pores (Figure 2a–c and Figure 3c 38 days of storage; Figure 4c pH 6.4; Figure 6(b3) and Figure 7e), and to the growth and fusion of membrane surface defects (Figure 1(a2) and Figure 3b 16–24 days of storage; Figure 4b pH 6.4; Figure 6(a2)).

The development of membrane surface nanodefects was similar to the dynamics of large cytoskeleton pores. These relationships are shown in Figure 7f. On the left, there are pores in the cytoskeleton network; on the right, there is their appearance on the membrane nanosurface. Obviously, the connections shown in Figure 7f are not dominant manifestations of the connections between cytoskeletal pores and grain domains on the membrane surface. Such an accurate experimental reproduction of a given cytoskeleton pore for an exactly given nanodefect of the membrane surface is practically impossible with instrumental methods. For this reason, statistical approaches are used for the comparison. The sizes and configurations of large cytoskeleton pores formed as a result of the factors shown in this work are close to the parameters of the domains on the nanosurfaces of RBC membranes (Figure 7b–e). Thus, the size of a large cytoskeleton pore (Figure 7e) was 559 nm, similar to the size of a domain formed from a group of grains (Figure 7c). The configuration and size of the pores in Figure 7b,c are close to each other.

## 3. Discussion

**Figure 8:**
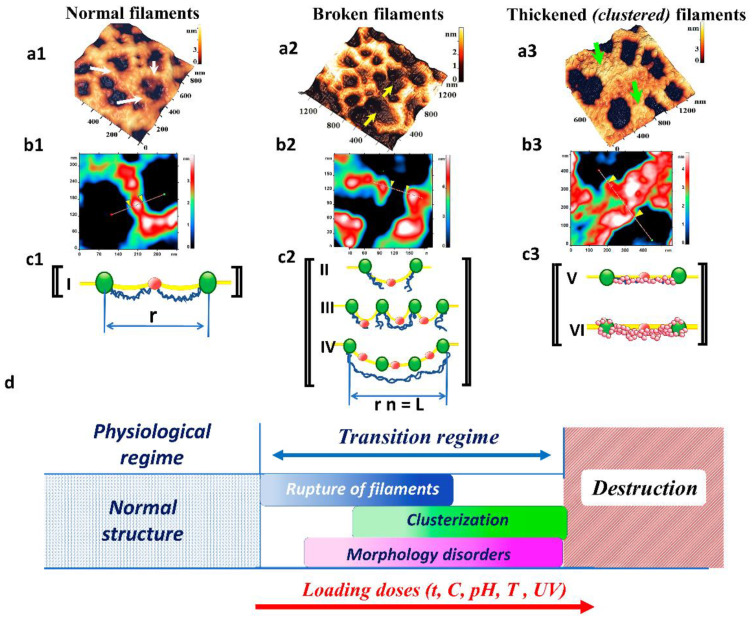
Stages of the RBC cytoskeleton transformation: (**a**) 3D AFM images of cytoskeleton fragments. (**b**) AFM images of cytoskeleton fragments, II order of the spatial Fourier transformation. (**c**) Schematic representation of processes. (**a1**–**c1**) Fragment of normal filaments of cytoskeleton network, white arrows. (**a2**–**c2**) Broken filaments, yellow arrows. (**a3**–**c3**) Clustered filaments, green arrows. The boundaries of single filaments in (**b1**–**b3**) are shown by markers. Size scales and color scales of heights are indicated for AFM images. (**c**)—green dots are ankyrin, red dots are actin complexes, and yellow lines are lipid bilayer. (**d**)—processes of filament rupture, their clustering, and disorders of RBC morphology as a function of summarized loading dose, the brightness of color in the transition regime corresponds to the intensity of the represented processes.

The cytoskeleton network consists of an ensemble of filaments [32]. AFM images show that each one can exist as a single thin filament (Figure 8(a1–c1)), as a broken filament (Figure 8(a2–c2)), and as a thickened clustered filament (Figure 8(a3–c3)). The thickness of a single filament (network septum) consisting of individual protein granules was 35–50 nm (Figure 8(b1)). In all experiments, two subsequent phenomena were recorded with a loading dose increase–hemin concentration, time of pRBCs storage, decrease in pH value, increase in suspension temperature and increase in UV irradiation dose. It was observed, first, filament network rupture (Figure 8(a2,b2)) and pore enlargement, and then the clustering and thickening of filaments (Figure 8(a3,b3)). Clustered thickened filaments consisted of grouped layers of individual granules and were on average 200–300 nm thick. These processes were common to all types of the indicated exposures and had similar patterns. They were ultimately caused by oxidative stress and precisely by ROS [33].

### 3.1. Filament Ruptures

During oxidation, spectrin-membrane linkage arises, leading to dissociation of a large proportion of the tetramers into dimers, filament rupture occurs [34]. We show these states by AFM images (Figure 8(a2–c2) II, III, IV). Rupture of spectrin filaments (cytoskeleton filaments) is associated with the appearance of domains on membrane nanosurfaces under the influence of hemin, long-term storage of pRBCs, decrease in the pH of the solution, and action of UV. Similar topological defects in the membranes were observed during gamma-irradiation of cells before transfusion [35], during RBC storage [36], and during the action of coral proteins on blood [37].

### 3.2. Clustering of Protein Components

The dissociation of spectrin tetramers may lead to translational diffusion of transmembrane proteins resulting in cluster formation and binding to membrane skeletal constituent [34]. The high-molecular-weight aggregates of proteins may be bound to the membrane [38]. By AFM, we showed clustered cytoskeleton filaments (Figure 8(a3–c3) V, VI). During oxidation, Hb degradation and stronger binding of degraded Hb to the membrane occur [39]. A loss of binding between band 3 and the cytoskeleton may be responsible for the storage-related increase in band 3 oligomerization and/or aggregation [40], as well as for increased susceptibility to crosslinking [40,41,42]. After Hb auto-oxidizes to metHb, it denatures to hemichromes, which then precipitate onto the lipids and the cytoskeleton of the RBC membrane [43]. The aggregation process may be regarded as a first-order phase transition. Thus, ROS causes oxidation of proteins and formation of protein-protein cross-linked derivatives.

Large cytoskeleton pores are formed as a result of filament ruptures (spectrin filaments). A single filament tear generates one double pore in size (Figure 8(c1,c2)). Hence, for the linear case, the size of a large pore *L*:

(1) L=r(n+1)q,

where *r* is the size of a single normal cytoskeleton pore, *n* is the number of filament ruptures, *q* is the coefficient taking into account the configuration of a large pore. So, in linear approximation, the size of large pores formed as a result of filament ruptures is a multiple of their normal size. The formation of cytoskeleton pores of size *L* (Equation (1)) produces corresponding changes on the surface of the membrane lipid bilayer in the form of grain domains. The number of grains in the domain on the membrane surface depends on the number of united merged elementary cytoskeleton pores. The processes of large pore formation (number *N* and size *L*) in the cytoskeleton and the appearance and fusion of nanodefects on membrane surfaces proceed simultaneously and develop both in time and in plane space (*x*, *y*). They depend on the loading dose (*E*) (hemin concentration, intensity of oxidative processes during storage of pRBCs, pH value and temperature of RBC suspension in buffer solution, UV irradiation dose), on time of the mentioned processes (*t*), and on local conditions in cytoskeleton and lipid bilayer membrane structures (*U*).

(2) N, L (t, x, y)=(n+1)rqf(t, E, U)

Dependences of filament rupture and their clustering processes and further disorders of RBC morphology on generalized loading doses are shown in Figure 8d.

These processes occur at doses higher than physiological ones and are realized in the transition regime. The intensity of these processes increases with the load (color brightness in Figure 8d). At low loads, filament ruptures begin. It causes changes in morphology and clustering of cytoskeleton filaments as loading increases. Large loading dose on RBCs lead to their destruction (e.g., temperature 49–50 °C, Figure 5).

Thus, the influence of factors of a physical and biochemical nature causes structural rearrangements in RBCs at all levels of organization, forming a unified mechanism of disturbances in relationships “cytoskeleton–membrane nanosurface-cell morphology”.

Our study has limitations. The presented studies were carried out in model experiments in vitro and cannot be unambiguously transferred to the clinical level. The influence of the investigated factors on blood cells in human organisms in vivo requires additional studies.

## 4. Materials and Methods

### 4.1. Preparation of Blood Samples

Blood of 6 healthy donors (4 men and 2 women, 25 to 51 years old) was used for experiments on the influence of hemin, different pH, UV radiation, and temperature change of RBC suspension in buffer solution. Blood sampling (150 μL) was performed in microvettes with EDTA (Sarstedt AG & Co., Numbrecht, Germany) during a prophylactic examination. We received informed consent from each donor.

For the experiments of blood storage, we used leukoreduced packed red blood cells. The pRBCs units were stored for 40 days in CPD/SAGM solution at +4 °C. Hematocrit was 60–65%. A single unit of pRBCs was 450 mL in volume. The pRBC units were obtained from Moscow blood transfusion centers. Two units of two blood groups were used in the study: O (I), A (II). On the experimental day, the sample was taken from pRBCs units without violating hermetical seal and integrity.

Research protocol was approved by the Ethics Committee of the Federal Research and Clinical Center of Intensive Care Medicine and Rehabilitology, Moscow, Russian Federation (protocol no. 2/20 of 10 June 2020).

To obtain smears for atomic force microscope scanning, 10 μL of the sample was dropped onto a slide. The RBC monolayer was prepared using a V-Sampler device (Vision, Vienna, Austria). The sample was air-dried before scanning by AFM.

### 4.2. Preparation of the Cytoskeleton

First, 500 µL of hypotonic solution (1 part 0.9% NaCl and 9 parts distilled water) was added to 100 µL of RBCs. It caused hemolysis of RBCs. Subsequently, the obtained suspension was centrifuged at 400× *g* for 5 min by a Universal 320 centrifuge (Andreas Hettich GmbH & Co., KG, Tuttlingen, Germany). The supernatant was removed, and 75 µL of the suspension was left in the Eppendorf tube. In the next step, 300 µL of distilled water was added to 75 µL of sediment to continue hemolysis. This obtained suspension was stirred for 5 min on Mini-Rotator Bio RS-24 (SIA BIOSAN, Riga, Latvia) (8 rpm, 5 min) and left in the refrigerator at 4 °C for 30 min and then at room temperature for 10 min to continue hemolysis. The sample was centrifuged at 400× *g* for 5 min. The ghost monolayer was prepared from the sediment on a slide using a V-Sampler before scanning by AFM.

For experiments to prepare cytoskeleton during storage of pRBCs, it was necessary to remove the anticoagulant-preservative solution. To do this, 500 µL of phosphate buffer (PBS) pH 7.4 (MP Biomedicals, Illkirch-Graffenstaden, France) was added to 100 µL of pRBC suspension. This suspension was stirred on Mini-Rotator Bio RS-24 at 8 rpm for 5 min. The second step was separation. After this step, the suspension was centrifuged at 400× *g* for 5 min. The supernatant was removed, and 500 µL of buffer (pH 7.4) was added. This process was repeated 5 times. The cytoskeleton was obtained on pRBCS washed from the anticoagulant-preservative solution.

### 4.3. Hemin

Dry hemin (Sigma-Aldrich, Saint Louis, MO, USA) was used in the experiment. First, 200 mg NaOH was dissolved in 10 mL of distilled water. Next, 50 mg of dry hemin and 5 mL of distilled water were added to 1 mL of the NaOH solution prepared above. The obtained basic hemin solution was added to 120 μL of blood in a microvette in the concentration range of 1.2 to 3.0 mM. The exposure time was varied from 120 to 200 min.

### 4.4. Storage of pRBCs

For the experiments, the sample was taken from the pRBC bag on days 4, 16–24, and 38 of storage. The pRBCs were stored for 40 days at +4 °C according to the WHO recommendations.

### 4.5. Influence of Different pH

120 μL of blood was added to 1 mL of PBS with pH 7.4; 6.8; 6.4 (Immunotex LLC, Stavropol, Russia). The obtained samples were incubated for 24 h at 20 °C. Samples were taken for the study after 1, 4, 6, 8, 12, and 24 h.

### 4.6. Influence of UV Radiation

In this series of experiments, an ultraviolet bactericidal irradiator (ORUBp-3-3, KRONT, Khimki, Russia) was used. Wavelength was λ = 254 nm, photon energy E = 4.8 eV, energy flux Φ = 5.2 W. The irradiated area of the suspension in a Petri dish was S = 12 cm^2^. The distance from the irradiated layer to the lamp was 0.5 cm. We added 3 mL of PBS pH 7.4 to 1 mL of RBCs washed from the plasma and put on a magnetic stirrer to ensure homogeneity of the irradiation. Finally, 4 mL of the suspension was irradiated for 30 and 60 min.

### 4.7. Temperature Change of RBC Suspension in Buffer Solution

120 μL of blood was placed in Biosan TDB-120 (SIA BIOSAN, Riga, Latvia) thermostat and incubated for 30 min at the specified temperature. The temperature ranges were as follows: 30–37, 40–43, and 49–50 °C.

### 4.8. Atomic Force Microscopy

Atomic force microscope NTEGRA Prima (NT-MDT Spectrum Instruments, Moscow, Zelenograd, Russia) was used to obtain images of cells, their nanostructure, and cytoskeleton. Images were obtained using NSG01 cantilevers with gold reflective coating, 10 nm probe radius, and stiffness coefficient of 1.45–15.1 N/m (TipsNano, Tallinn, Estonia). Fields ranging in size from 100 × 100 to 2 × 2 μm^2^ were scanned in the semicontact mode. The number of points for each image was set from 512 to 1024.

### 4.9. Spatial Fourier Transform of Membrane Nanosurface and Cytoskeleton Nanostructure

Detailed analysis of membrane nanosurfaces was performed using spatial Fourier decomposition [3,44]. For this purpose, AFM software of Integra Prima SPM Nova PX software (NT-MDT Spectrum Instruments, Moscow, Zelenograd, Russia) and software product FemtoScan Online (Femtoscan, Moscow, Russia) were used. The choice of spectral windows of orders I and II was determined by the nanostructure of the erythrocyte membrane surface [44]. I order, spectral window of 600–1000 nm, II order, spectral window of 70–300 nm. Decomposition of the nanosurface into I and II orders made it possible to register individual protein fragments that are part of the cytoskeleton filaments of erythrocytes.

### 4.10. Statistical Analysis

#### 4.10.1. Cell Monolayer

When the indicated physical-chemical factors were applied to blood cell membranes, 3 areas of 100 × 100 μm^2^, 50 × 50 μm^2^ were scanned for each sample. A total of 600 images were scanned and analyzed. The number of scanning points was 512, 1024.

The nanostructure of erythrocytes of individual cells was obtained on 1.5 × 1.5 μm^2^ fragments. The number of scanning points was 1024. A total of 500 images of cell fragments were analyzed.

#### 4.10.2. Ghost Monolayer

To analyze the cytoskeleton structure, a 2.5 × 2.5 μm^2^ area was scanned in 10 ghosts for each sample. The number of scanning points was 1024. A total of 1000 sections in the ghosts were analyzed.

#### 4.10.3. Statistics

Statistical processing was performed using Origin Pro 2019 software (OriginLab Corporation, Northampton, MA, USA). All data are presented as the mean ± SD, histograms, and box charts. One-way ANOVA with post hoc. Tukey test was used for experimental data comparision.

## 5. Conclusions

In this work, we showed how disruption of the RBC cytoskeleton filaments generates large pores in it and how this process occurs on the membrane nanosurface in the form of grain domains. The results may be useful for understanding the role of cytoskeleton disruptions in the formation of membrane nanodefects, which in turn trigger changes in the RBC morphology.


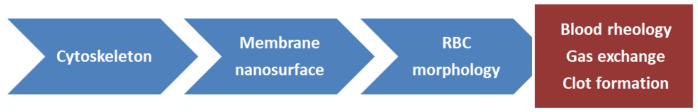


Since the development of these processes can lead to changes in the rheological properties of blood, impaired gas exchange, and vascular clot formation, the complex approach to study the molecular-structural mechanism of disorders in RBCs may be important in the diagnosis and treatment of human diseases.

The results of this research can be used in molecular cell biology, membrane biophysics, and in fundamental and practical medicine.

## Data Availability

The data sets used and analyzed during the current study are available from the corresponding authors on request.
